# Development and Validation of a Robust Ferroptosis-Related Gene Panel for Breast Cancer Disease-Specific Survival

**DOI:** 10.3389/fcell.2021.709180

**Published:** 2021-11-25

**Authors:** Pei Li, Benlong Yang, Bingqiu Xiu, Yayun Chi, Jingyan Xue, Jiong Wu

**Affiliations:** ^1^ Department of Breast Surgery, Fudan University Shanghai Cancer Center, Shanghai, China; ^2^ Department of Breast Surgery, Key Laboratory of Breast Cancer in Shanghai, Fudan University Shanghai Cancer Center, Shanghai, China; ^3^ Collaborative Innovation Center for Cancer Medicine, Shanghai, China

**Keywords:** breast cancer, ferroptosis, prognosis model, immune status, disease-specific survival

## Abstract

**Background**: New biomarker combinations have been increasingly developed to improve the precision of current diagnostic and therapeutic modalities. Recently, researchers have found that tumor cells are more vulnerable to ferroptosis. Furthermore, ferroptosis-related genes (FRG) are promising therapeutic targets in breast cancer patients. Therefore, this study aimed to identify FRG that could predict disease-specific survival (DSS) in breast cancer patients.

**Methods**: Gene expression matrix and clinical data were downloaded from public databases. We included 960, 1,900, and 234 patients from the TCGA, METABRIC, and GSE3494 cohorts, respectively. Data for FRG were downloaded from the FerrDb website. Differential expression of FRG was analyzed by comparing the tumors with adjacent normal tissues. Univariate Cox analysis of DSS was performed to identify prognostic FRG. The TCGA-BRCA cohort was used to generate a nine-gene panel with the LASSO cox regression. The METABRIC and GSE3494 cohorts were used to validate the panel. The panel’s median cut-off value was used to divide the patients into high- or low-risk subgroups. Analyses of immune microenvironment, functional pathways, and clinical correlation were conducted *via* GO and KEGG analyses to determine the differences between the two subgroups.

**Results**: The DSS of the low-risk subgroup was longer than that of the high-risk subgroup. The panel’s predictive ability was confirmed by ROC curves (TCGA cohort AUC values were 0.806, 0.695, and 0.669 for 2, 3, and 5 years respectively, and the METABRIC cohort AUC values were 0.706, 0.734, and 0.7, respectively for the same periods). The panel was an independent DSS prognostic indicator in the Cox regression analyses. (TCGA cohort: HR = 3.51, 95% CI = 1.792–6.875, *p* < 0.001; METABRIC cohort: HR = 1.76, 95% CI = 1.283–2.413, *p* < 0.001). Immune-related pathways were enriched in the high-risk subgroup. The two subgroups that were stratified by the nine-gene panel were also associated with histology type, tumor grade, TNM stage, and Her2-positive and TNBC subtypes. The patients in the high-risk subgroup, whose CTLA4 and PD-1 statuses were both positive or negative, demonstrated a substantial clinical benefit from combination therapy with anti-CTLA4 and anti-PD-1.

**Conclusion**: The new gene panel consisting of nine FRG may be used to assess the prognosis and immune status of patients with breast cancer. A precise therapeutic approach can also be possible with risk stratification.

## Introduction

Breast cancer (BRCA) has entered the era of precision treatment at the molecular level. The molecular hallmarks of BRCA, including ER, PR, HER2, Ki-67, and PD-1, and PD-L1, have been employed for personalized and individualized treatment (Harbeck et al., 2019; Waks and Winer, 2019). For instance, the endocrine therapy, HER2 targeted therapy, and immune checkpoint therapy are employed for ER/PR-positive, Her2-positive, and PD-1/PD-L1-positive BRCA tumors, respectively^2^.

Since the advancement of microarray and high-throughput sequencing, multi-gene prediction, such as the PAM50 signature (Cheang et al., 2015), 70-gene assay, and 21-gene recurrence score^2^, has been widely used to guide decision making in the therapeutic approach for various BRCA subtypes. Multi-gene prediction has been commonly used to predict the benefits of chemotherapy or to estimate patient’s prognosis. Thus, new biomarker combinations have been developed to improve the precision of current diagnostic and therapeutic modalities. Currently, researchers are looking for new therapeutic targets and biomarkers for BRCA treatment. Moreover, there is still an insurmountable therapeutic challenge for triple negative breast cancer (TNBC) because therapeutic targets and biomarkers have not yet been identified. A few previous studies found that several ferroptosis-related genes (FRG) could be promising therapeutic targets in BRCA (Hangauer et al., 2017; Zhang et al., 2019), especially for the TNBC subtype (Chen et al., 2017; Zhu et al., 2020; Ding et al., 2021; Zhang et al., 2021). Therefore, it is necessary to develop a panel involving FRG biomarkers for risk stratification and identification of new targets.

Ferroptosis refers to cell death resulting from iron-mediated lipid peroxidation and is characterized by intracellular accumulation of reactive oxygen species (ROS) (Supplementary Figure S3) (Dixon et al., 2012). According to preliminary data, ferroptosis inhibits tumor development and proliferation; hence, ferroptosis can be targeted for cancer therapy (Stockwell et al., 2017). Correspondingly, researchers have increasingly focused on the role of ferroptosis in BRCA, especially in TNBC and Her2-positive BRCA. Due to the high recurrence and metastatic rate, TNBC and Her2-positive BRCA have been regarded as refractory and aggressive BRCA subtypes (Foulkes et al., 2010; Cesca et al., 2020). Ma et al. (Dixon et al., 2012) found that siramesine and lapatinib induced ferroptosis more than other canonical ferroptotic reagents did, implying that lapatinib participated in modulating ferroptosis without targeting EGFR and HER2 (Ma et al., 2016). Moreover, a recent study discovered that neoadjuvant neratinib induced ferroptosis and prevented brain metastasis in Her2-positive BRCA (Nagpal et al., 2019). Furthermore, several studies reported that ferroptosis could be a useful therapeutic target in the treatment of TNBC (Chen et al., 2017; Zhu et al., 2020; Ding et al., 2021; Zhang et al., 2021). It has been found that through the stimulated GCN2-eIF2-ATF4 pathway, CHAC1 degradation of GSH increases cystine-starvation-induced ferroptosis in TNBC cells (Chen et al., 2017). The anti-TNBC impact of DMOCPTL was demonstrated in a cell death method test by triggering ferroptosis *via* GPX4 ubiquitination (Ding et al., 2021). Chen et al. found that treatment of TNBC cells with holo-Lf increased total iron concentration, boosted ROS production, increased the lipid peroxidation end product malondialdehyde, and improved ferroptosis (Zhang et al., 2021). In addition, chemotherapy, radiotherapy, and immunotherapy were all influenced by ferroptosis; thus, targeting both ferroptosis and the identified biomarkers could be an effective treatment strategy for BRCA (Chen et al., 2021).

The aim of this research was to develop a panel consisting of FRG that could be used to predict the disease-specific survival (DSS) of patients with BRCA and to develop a risk stratification system that could aid diagnosis and provide novel therapeutic strategies.

## Materials and Methods

**FIGURE 1:**
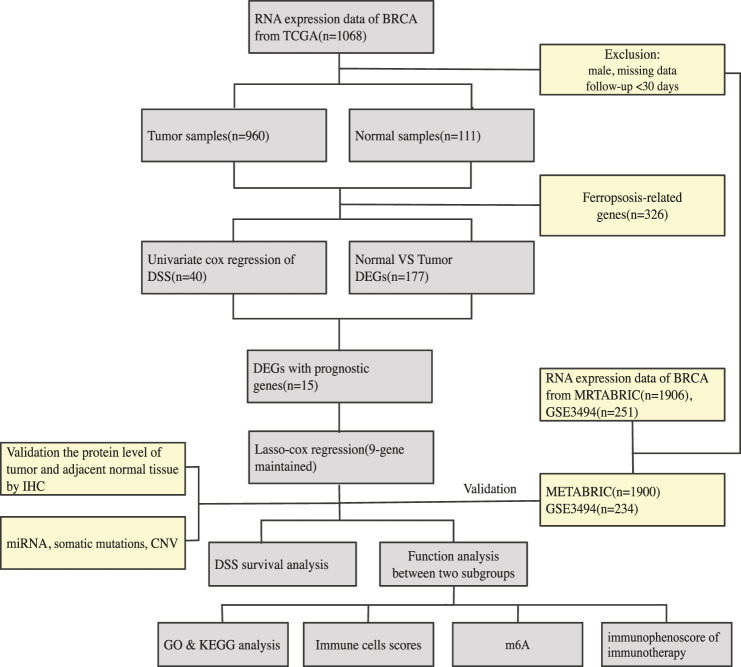
An overview of our methodology is summarized.

An overview of our methodology is summarized in Figure 1.

### Acquisition of the Gene, miRNA, and Genome Mutation Data; FRG; and Clinical Data

On January 30, 2021, 1,068 patients with BRCA were identified. Their gene, miRNA, genome mutation data (containing somatic mutations and copy number variations (CNV)), and clinical information were downloaded from the TCGA website. On the same day, the gene expression matrix and clinical data of 1,906 patients with BRCA were downloaded from the cBioPortal website. The patients whose data were obtained from both websites were the same patients involved in the Molecular Taxonomy of BRCA International Consortium (METABRIC) project. Since the data from both websites were open to the public, this study was exempted from obtaining the approval of the local ethics committee. The GSE3494 dataset were downloaded from the Gene Expression Omnibus (GEO) database. The current study adhered to the TCGA, METABRIC, and GEO data access and publishing policies. The exclusion criteria were as follows: male sex, incomplete clinical and gene expression data, and less than 30 days of DSS follow-up. Finally, we included 960, 1,900, and 234 patients from the TCGA, METABRIC, and GSE3494 cohorts, respectively. The baseline features of the three cohorts are presented in Table 1.

**TABLE 1:** The baseline features of the TCGA, METABRIC and GSE3494 cohorts.

Characteristics	TCGA	METABRIC	GSE3494
Number	n = 960	n = 1900	n = 234
Age(average)	58	61.1	62.7
Race(%)			
White	666 (69.4)		—
Black	168 (17.5)	—	-
Asian and other	52 (5.4)	—	—
NA	74 (7.7)	—	—
Tumor grade(%)			
G1	—	164 (8.6)	62 (2.5)
G2	—	740 (38.9)	120 (51.3)
G3	—	925 (48.7)	50 (21.4)
NA	—	71 (3.7)	2 (0.9)
Histological type(%)			
IDC	704 (73.3)	1450 (76.3)	—
ILC	191 (19.9)	142 (7.5)	—
Other	65 (6.8)	308 (16.2)	
Menopause status(%)			
Pre	209 (21.8)	411 (21.6)	—
Post	616 (64.2)	1489 (78.4)	—
Peri	37 (3.9)	—	—
NA	98 (10.2)	—	—
ER status (%)			
Positive	716 (74.6)	1457 (76.7)	200 (87)
Negative		443 (23.3)	30 (13)
NA	41 (4.3)		—
PR status (%)			
Positive	624 (65)	1007 (53)	178 (76.1)
Negative		893 (47)	56 (23.9)
NA	43 (4.5)	0	—
HER2 status (%)			
Positive	169 (17.6)	236 (12.4)	—
Negative	672 (70)	1667 (87.7)	—
NA	122 (12.7)	0	—
Molecular subtyp**e**			
HR+/Her2-	535 (56)	1378 (73)	—
HR+/Her2+	132 (14)	104 (5)	—
HR-/Her2+	33 (3)	132 (7)	—
TNBC	135 (14)	299 (16)	—
NA	125 (13)	—	—
TNM stage(%)			
0	—	4 (0.2)	—
I	166 (17.3)	473 (24.9)	—
II	545 (56.8)	800 (42.1)	—
III	211 (22)	115 (6.1)	—
IV	18 (1.9)	9 (0.5)	—
NA	20 (2.1)	499 (26.3)	—
follow up state(%)			
alive	883 (92)	1279 (67.3)	180 (76.9)
dead	77 (8)	621 (32.7)	54 (23.1)
DSS years (median)	2.5	9.5	10.2

IDC: invasive ductal carcinoma, ILC: invasive lobular carcinoma.

The list of FRG was downloaded from the FerrDb website. The website divided FRG into three categories: drivers, which stimulate ferroptosis, suppressors, which prevent ferroptosis, and biomarkers, which reveal the presence of ferroptosis (Zhou and FerrDb, 2020). We reviewed the existing literature to identify these genes, and we subsequently ruled out unrelated genes and added newly discovered genes that were related to this study. The immunohistochemistry (IHC) image data of prognostic ferroptotic proteins were downloaded from the Human Protein Atlas (HPA) database.

### Identification of Differentially Expressed and Prognostic Genes

Running the “Limma” R package, the TCGA cohort was used to identify differently expressed FRG by comparing the expression levels in tumor and adjacent normal tissues (log FC > 0.5, FDR <0.05). We subsequently used univariate Cox analysis of DSS to identify prognostic FRG. For survival outcomes, DSS event was defined as death due to BRCA, while no event was defined as death due to causes other than BRCA or a living status. The intersect gene set was identified as the FRG that were both differentially expressed and prognostic. The LASSO process was used to pick and shrink the important variables in the regression panel by running the “glmnet” R package (Tibshirani, 1997; Wang and Liu, 2020). The DSS statuses of the TCGA cohort patients were the response variables in the regression, with the matrix of the intersect gene set as the independent variable. The panel’s penalty parameter was calculated using the cross validation, which was multiplied by ten, and the optimal parameter was the λ value that corresponded to the lowest deviation. The patients’ risk scores were calculated using each of the selected gene expressing values, which were multiplied by their coefficients. The formula was as follows:

$$\begin{array}{l} the\;risk\;score\;formula\;\\ \;\;\;\;\;=\;coefficients\;*\;expressing\;values\;of\;A\;gene+\;coefficients\\ \;\;\;\;\;*expressing\;values\;of\;B\;gene\ldots \;\ldots + \end{array}$$

The patients were classified into the high- or low-risk subgroups according to the median cut-off value of the developed panel. PCA and t-SNE were used to investigate the distribution of the two subgroups by running the “Rtsne” R package. The optimal cut-off expressing values for each gene were determined by running the “survminer” R package. The “ggalluvial” R package was used to portray the Sankey map. The predictive ability of the developed panel was determined by time-dependent receiver operating characteristic (ROC) curves by running the R “timeROC” package. The area under curve (AUC) of the ROC curve was determined to show the sensitivity and specificity of the panel in providing a prognostic efficiency, which varied from 0.5–1. Values that were closer to 1 indicated a good prognostic ability.

To establish the miRNA-FRG regulatory network in BRCA. We put the FRG into the starBase database to identify potential miRNAs (Li et al., 2013). We then conducted an analysis in TCGA-BRCA and adjacent normal tissue to identify the different miRNAs. In addition, we sought candidate miRNAs that were only shared by the two databases to enhance the veracity of the prediction. Finally, the network was visualized using Cytoscape. (Supplementary Figure S2).

The “maftools” and “Rcircos” R packages were used for somatic mutations identification and CNV, respectively.

We looked for publications on m6A methylation regulators in the literature and found 23 of them, with 8 writers (METTL3, METTL14, METTL16, RBM15, RBM15B, WTAP, ZC3H13, VIRMA), 2 erasers (FTO and ALKBH5), and 13 readers (YTHDC1, YTHDC2, YTHDF1, YTHDF3, HNRNPC, FMR1, IGFBP3, RBMX, IGFBP1, YTHDF2, HNRNPA2B1, LRPPRC, and KIAA1429).

### Function Enrichment Analysis, single-sample Gene Set Enrichment Analysis (ssGSEA), and immunophenoscore (IPS)

Based on the differentially expressed genes between the two stratified subgroups, Gene Ontology (GO) and Kyoto Encyclopedia of Genes and Genomes (KEGG) analyses were performed by running.

the “clusterProfiler” R package. Differentially expressed genes between the high- and low-risk subgroups were identified (|log2FC| > 1, FDR <0.05). The Benjamini-Hochberg (BH) method was used to adjust the *p* values.

The “GSVA” R package was used to measure the infiltrating score of 16 immune cells and the activity of 13 immune-related pathways with ssGSEA (Rooney et al., 2015). The Wilcoxon test was also used to look at intergroup variations in putative immunological checkpoints, such as PD-L1, PD-1, and CTLA4. Furthermore, to predict the efficacy of immunotherapy, we downloaded an IPS file of immune checkpoint inhibitors (ICIs) from the Cancer Immunome database; the IPS is a good predictor for responsiveness to CTLA4 and PD-1, and predicts the intergroup differences in response to immunotherapy using CTLA4 and PD-1 blockers (Charoentong et al., 2017).

### Statistical Analyses

The gene matrix of tumor and adjacent normal tissues was compared using the Student’s *t*-test. The Χ^2^ test was used to compare the proportional differences. The ssGSEA scores of immune cells or pathways were compared between the high- and low-risk subgroups using the Mann-Whitney test with *p* values that were adjusted by the BH method. The log-rank test was used to compare the DSS of different subgroups using the Kaplan-Meier analysis. The univariate and multivariate Cox regression tests were used to identify independent predictors of DSS. The R program (version 3.6.3) or Statistical Package for the Social Sciences (SPSS), version 20 was used for all statistical analyses. A *p* value of less than 0.05 was deemed statistically significant, unless otherwise stated, and all *p* values were two-tailed.

## Results

### Identification of 177 Differentially Expressed FRG and 40 DSS Prognostic FRG

**FIGURE 2:**
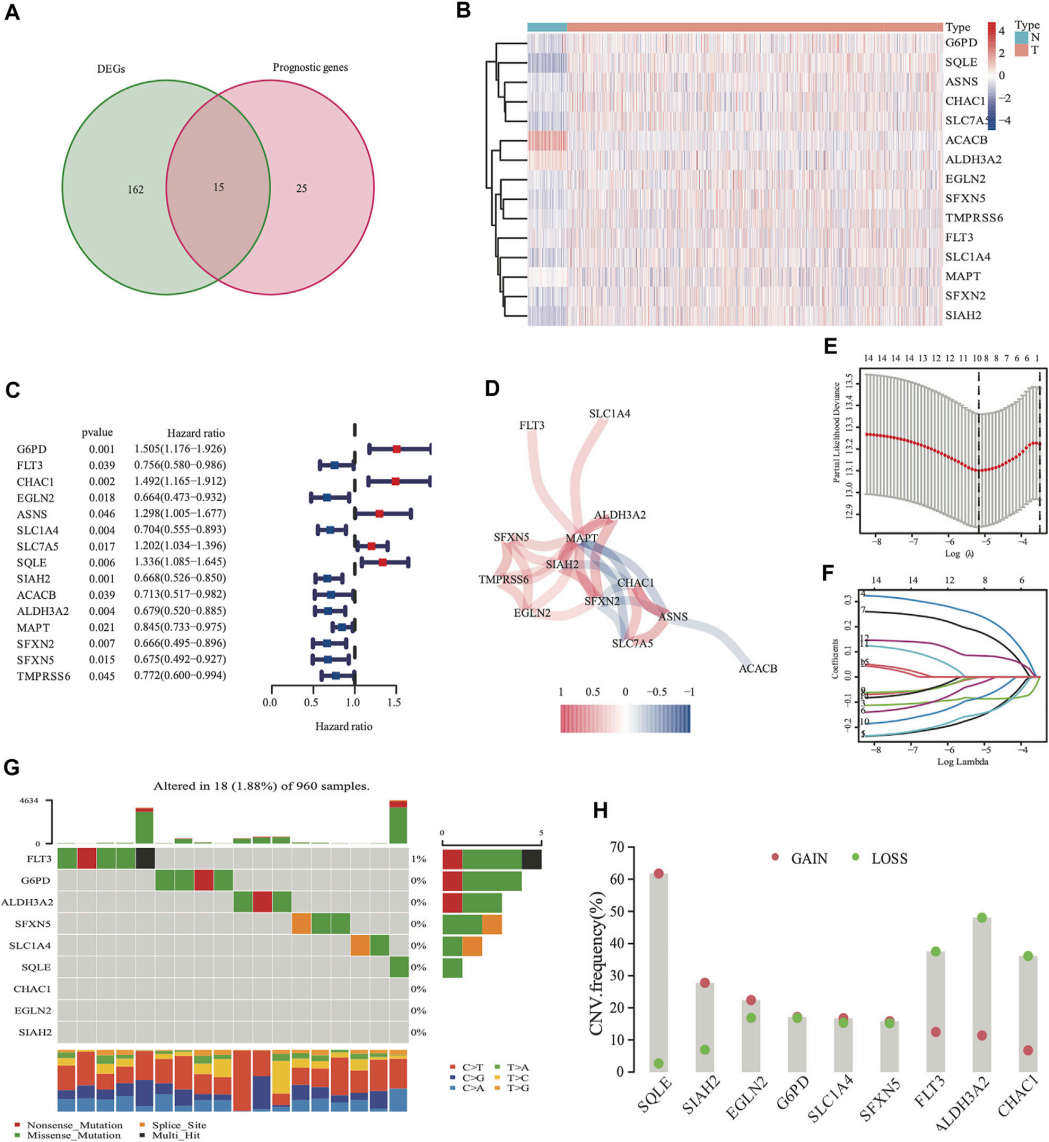
Identification of 177 differentially expressed genes (DEGs) and 40 DSS prognostic FRG. A venn diagram was used to identify overlapping genes **(A)**. Heatmap showed that 15 FRG were differentially expressed in breast cancer tissues and non-cancer tissues **(B)** Forest plots demonstrated the hazard ratio of the univariate cox analyses in DSS **(C)**. The correlation link of the selected genes in TCGA cohort **(D)**. Lasso regression analysis reduced variable **(E, F)**. Somatic mutations on a query of FRG from TCGA cohort **(G)** The CNV frequency of FRG from the TCGA cohort **(H)**, red dots represent CNV amplification, while green dots represent CNV deletion.

There were 177 FRG, which were differentially expressed between the tumor and adjacent normal tissues (all FDR <0.05, log FC > 0.5, Figure 2A). In addition, there were 40 FRG, which were associated with DSS (Figure 2A) in the univariate Cox regression study. Moreover, 15 genes were maintained in the intersection of the 177 differentially expressed and 40 prognostic FRG genes. Most of them were upregulated, except for the following genes: *ACACB* and *ALDH3A2* (Figure 2B). *CHAC1, SIAH2, MAPT, SFXN2*, and *ASNS* were identified to be the hub genes in the interaction network among these genes. Figure 2D shows the relationship among these genes.

### Development of a Prognostic Panel With the TCGA Cohort, and Analysis of Nine FRG Genome Mutations

The expression values of the 15 overlapped genes were used to create a prognostic panel with the LASSO regression analysis. The following formula was used to measure the risk score. The optimum value of λ was used to identify a nine-gene signature (Figures 2E,F).

$$\begin{array}{l} =risk\;score=0.181*expression\;values\;of\;\left(EV\right)SQLE+0.247*EV\;of\;G6PD\;+\;0.085\\ \;\;\;\;\;*EV\;of\;CHAC1-0.158*EV\;of\;ALDH3A2-0.086*EV\;of\;SIAH2-0.149\\ \;\;\;\;\;*EV\;of\;SLC1A4-0.026*EV\;of\;FLT3-0.001*EV\;of\;EGLN2-0.084\\ \;\;\;\;\;*EV\;of\;SFXN5 \end{array}$$

**FIGURE 3:**
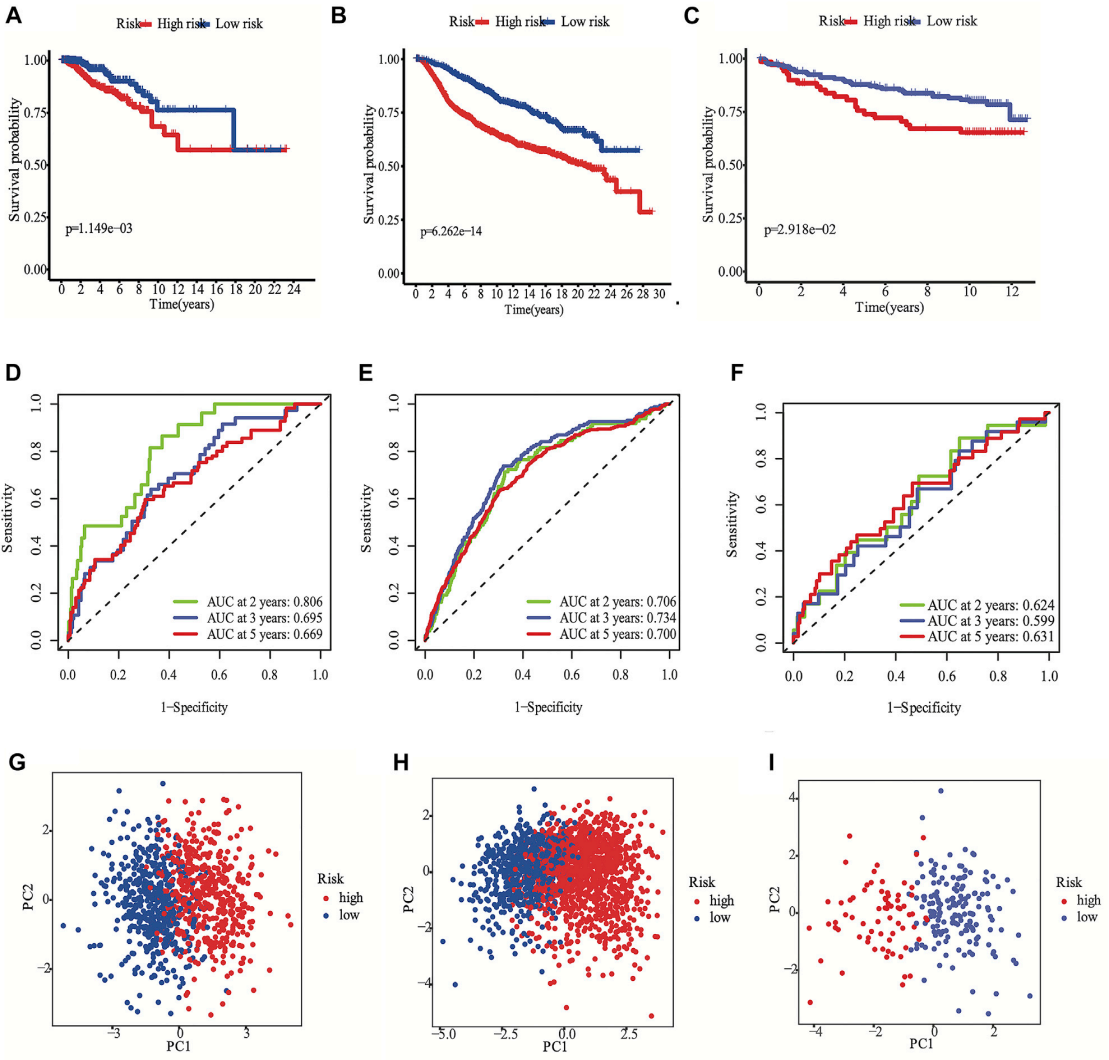
Development the prognostic FRG panel in TCGA cohort **(A, D, G)**. Validation the prognostic FRG panel in METABRIC cohort **(B, E, H)** and GSE3494 cohort **(C, F, I)**. The Kaplan-Meier curve showed that the DSS of the high-risk subgroup was substantially shorter than that of the low-risk subgroup **(A, B, C)**. AUC time-dependent ROC curves for DSS **(D, E, F)**.

**FIGURE 4:**
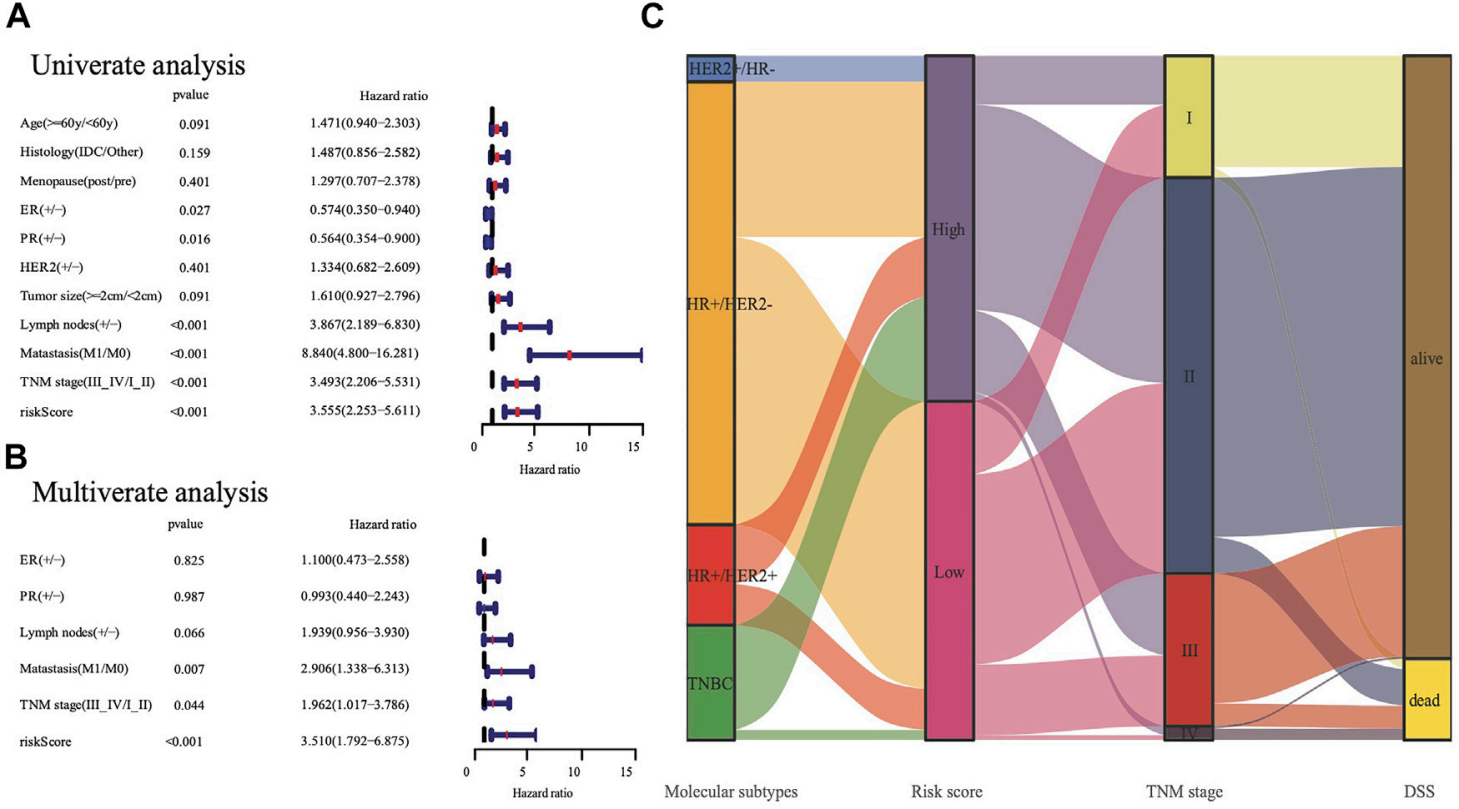
The analysis nine gene panel of FRG based on TCGA. The hazard ratio of univariate and multivariate Cox regression analysis **(A,B)**, (+/ -) means (positive/negative). A Sankey map demonstrated the relationship between FRG risk score and molecular subtypes **(C)**.

According to the median cut-off value of the nine-gene panel, patients were classified into the high- (n = 480) and low-risk (n = 480) subgroups. In the TCGA cohort, the risk score was associated with the histological type; HER2, ER, and PR statuses; TNM stage, and molecular subtype (Table 2). Moreover, the high-risk subgroup was positively associated with an advanced TNM stage and Her2-positive and TNBC subtypes (Table 2; Figure 4C). All of these indicators were related to an unfavorable prognosis in BRCA patients (Waks and Winer, 2019). The PCA and t-SNE showed that the patients of these two subgroups were distributed in two directions (Figure 3G and Supplementary Figure S1G). The risk score of patients were positively associated with a higher death toll (Supplementary Figures S1A, 1D). The Kaplan-Meier curve consistently showed that the DSS of the high-risk subgroup was substantially shorter than that of the low-risk subgroup (Figure 3A, *p* = 0.001). The DSS predictive performance of the nine-gene panel was assessed by ROC curves, and the values of the AUC were 0.806, 0.695, and 0.669 in 2, 3, and 5 years, respectively, in the TCGA cohort (Figure 3D). The IHC staining results also provided the levels of seven (including *G6PD, SQLE, CHAC1, ALDH3A2, SIAH2, SLC1A4*, and *SFXN5*) of nine prognostic ferroptotic proteins between BRCA and adjacent normal tissues, consistent with the mRNA expression data (Figure 7).

**TABLE 2:** Clinical features in different risk subgroups.

Characteristics	TCGA-BRCA			METABRIC-BRCA			GSE3494-BRCA		
	High risk	Low risk	*p* Value	High risk	Low risk	*p* Value	High risk	Low risk	*p* Value
number(%)	960			1900			234		
	480 (50)	480 (50)		1252 (65.9)	648 (34.1)		70 (30)	164 (70)	
Age (%)			0.003			<0.001			0.309
<60y	283 (29.5)	237 (24.7)		598 (31.5)	241 (12.7)		31 (13.2)	61 (26.1)	
≥60y	197 (20.5)	243 (25.3)		654 (34.4)	407 (21.4)		39 (16.7)	103 (44)	
Race(%)			<0.001						
White	289 (30.1)	377 (39.3)	—	—	—		—	—	
Black	117 (12.2)	51 (5.3)		—			—	—	
Asian and other	36 (3.7)	16 (1.7)		—	—		—	—	
NA	38 (4)	36 (3.8)		—	—		—	—	
Tumor grade(%)			—			<0.001			<0.001
G1	—	—		54 (2.8)	110 (5.8)		7 (3)	55 (23.5)	
G2	—	—		370 (19.5)	370 (19.5)		28 (12)	92 (39.3)	
G3	—	—		788 (41.5)	137 (7.2)		35 (15)	15 (6.4)	
NA	—	—		40 (2.1)	31 (1.6)		—	2 (0.9)	
Histological type(%)			<0.001			<0.001			
IDC	430 (44.8)	274 (28.5)		1028 (54.1)	422 (22.2)		—	—	
ILC	28 (2.9)	163 (16.9)		64 (3.4)	78 (4.1)		—	—	
Other	22 (2.2)	43 (4.5)		161 (8.4)	148 (7.8)		—	—	
Menopause status(%)			0.723			<0.001			
Pre	106	103		301 (15.8)	110 (5.8)		—	—	
Post	302	314		951 (50.1)	538 (28.3)		—	—	
Peri	18	19		—	—		—	—	
NA	54	44		—	—		—	—	
ER status(%)			<0.001			<0.001			<0.001
Positive	274 (28.5)	442 (46)		814 (42.8)	643 (33.8)		51 (22.2)	149 (93.1)	
Negative	188 (19.6)	15 (1.6)		438 (23.1)	5 (0.3)		19 (8.3)	11 (4.8)	
NA	18 (1.9)	23 (2.4)		—	—				
PR status(%)			<0.001	—	—	<0.001			<0.001
Positive	224 (23.3)	400 (41.7)		496 (26.1)	511 (26.9)		40 (17.1)	138 (59)	
Negative	239 (24.9)	54 (5.6)		756 (39.8)	137 (7.2)		30 (12.8)	26 (11.1)	
NA	17 (1.78)	26 (2.7)		—	—		—	—	
HER2 status(%)			<0.001	—	—	<0.001			
Positive	114 (11.9)	55 (5.7)		227 (11.9)	9 (0.5)		—	—	
Negative	306 (31.9)	366 (38.1)		1027 (54.1)	640 (33.7)		—	—	
NA	63 (6.6)	59 (6.1)					—	—	
Molecular subtype			<0.001			<0.001			
HR+/Her2-	182 (19)	353 (60)		732 (38.5)	646 (33.4)		—	—	
HR+/Her2+	77 (16)	55 (11.5)		96 (7.7)	8 (1.2)		—	—	
HR-/Her2+	33 (3.4)	0		131 (6.9)	1 (0.1)		—	—	
TNBC	123 (12.8)	12 (1.3)		295 (15.5)	4 (1.3)		—	—	
NA	65 (6.8)	60 (12.5)		—	—		—	—	
TNM stage(%)			0.045			<0.001			
0				3 (0.2)	1 (0.1)		—	—	
I	67 (7)	99 (10.3)		263 (13.8)	210 (11.1)		—	—	
II	279 (29.1)	266 (27.7)		560 (29.5)	240 (12.6)		—	—	
III	113 (11.8)	98 (10.2)		98 (5.2)	17 (0.9)		—	—	
IV	12 (1.3)	6 (0.6)		7 (0.4)	2 (0.1)		—	—	
NA	9 (0.9)	11 (1.1)		321 (16.9)	178 (9.4)		—	—	
Follow up state(%)									
Alive	428 (44.6)	455 (47.4)		776 (40,8)	503 (26.5)		48 (20.5)	132 (56.4)	
Dead	52 (5.4)	25 (2.6)		476 (25.1)	145 (7.6)		22 (9.4)	32 (13.7)	
DSS years (median)	2.4	2.5		8.7	10.6		9.9	10.4	

#### Analysis of Nine FRG Genome Mutations and Establishing the miRNA-Nine-FRG Regulatory Network

The somatic mutations and CNV of nine FRG in breast cancer were initially summarized. Only 18 of the 960 patients (1.88%) had mutations in the nine FRG, and the mutation frequencies were zero in eight of nine except *FLT3* (1%; Figure 2G). We found that higher frequencies of CNV deletions were in *ALDH3A2, FLT3* and *CHAC1*; conversely, higher probabilities of CNV amplification were in *SQLE, SIAH2* and *EGLN2* (Figure 2H). Cytoscape showed that the network contains four hub miRNAs (hsa-miR-23a-3p, hsa-miR-378a-3p, hsa-miR-146a-5p, and hsa-miR-146b-5p). (Supplementary Figure S2).

### Validation of the Nine-Gene Panel With the METABRIC and GSE3494 Cohorts

The METABRIC and GES3494 cohorts were used to robustly validate the nine-gene panel using the same formula as that used for the construction of the panel using the TCGA cohort. Correspondingly, the nine-gene panel was also associated with the histological type, tumor grade; HER2, ER, and PR statuses; and TNM stage (Table 2). Similarly, the high-risk subgroup was positively associated with a high tumor grade; an advanced TNM stage; and Her2-positive and TNBC subtypes (Table 2). All of the above indicators were related to an unfavorable prognosis in patients with BRCA (Table 2). The death toll of the high-risk subgroup was also more than that of the low-risk subgroup (Figures 3B,C). The PCA and t-SNE analyses also indicated that the two subgroups were spread in discrete directions, which was consistent with the findings obtained from the TCGA cohort (Figures 3H,I and Supplementary Figures S1H, I). Patients in the high-risk subgroup consistently died from the tumor much sooner than those in the low-risk subgroup (Figures 3B,C), as determined by the Kaplan-Meier curve. Furthermore, the AUC of the ROC curve for the nine-gene panel was 0.706 after 2 years, 0.734 after 3 years, and 0.7 after 5 years in the METABRIC and were respectively 0.624, 0.599, and 0.631 in the GSE3494 cohort (Figures 3E,F).

### The Nine-Gene Panel has an Independent Prognostic Significance

We conducted univariate and multivariate Cox analyses to test if the nine-gene panel was an independent predictor of DSS. The univariate Cox regression analysis was conducted to reveal obvious linkages between the nine-gene panel and DSS in both the TCGA and METABRIC cohorts (TCGA cohort: HR = 3.555, 95% confidence interval [CI] = 2.253–5.611, *p* < 0.001; METABRIC cohort: HR = 2.511, 95% CI = 2.075–3.04, *p* < 0.001; Figure 4A). After controlling other confounding variables in the multivariate cox regression study, the nine-gene panel remained an independent indicator of DSS (TCGA cohort: HR = 3.51, 95% CI = 1.792–6.875, *p* < 0.001; METABRIC cohort: HR = 1.76, 95% CI = 1.283–2.413, *p* < 0.001) (Figure 4B). The panel had a high predictive accuracy for DSS and was even better than the statuses of ER, PR, HER2, tumor size, and lymph nodes. It offered a more reliable predictor of 2 years DSS in the TCGA (AUC = 0.806) and the METABRIC cohorts (AUC = 0.706) (Figures 3D,E). As a result, the panel had an outstanding prognostic benefit for patients with BRCA.

### Functional Studies Between the Stratified Subgroups

**FIGURE 5:**
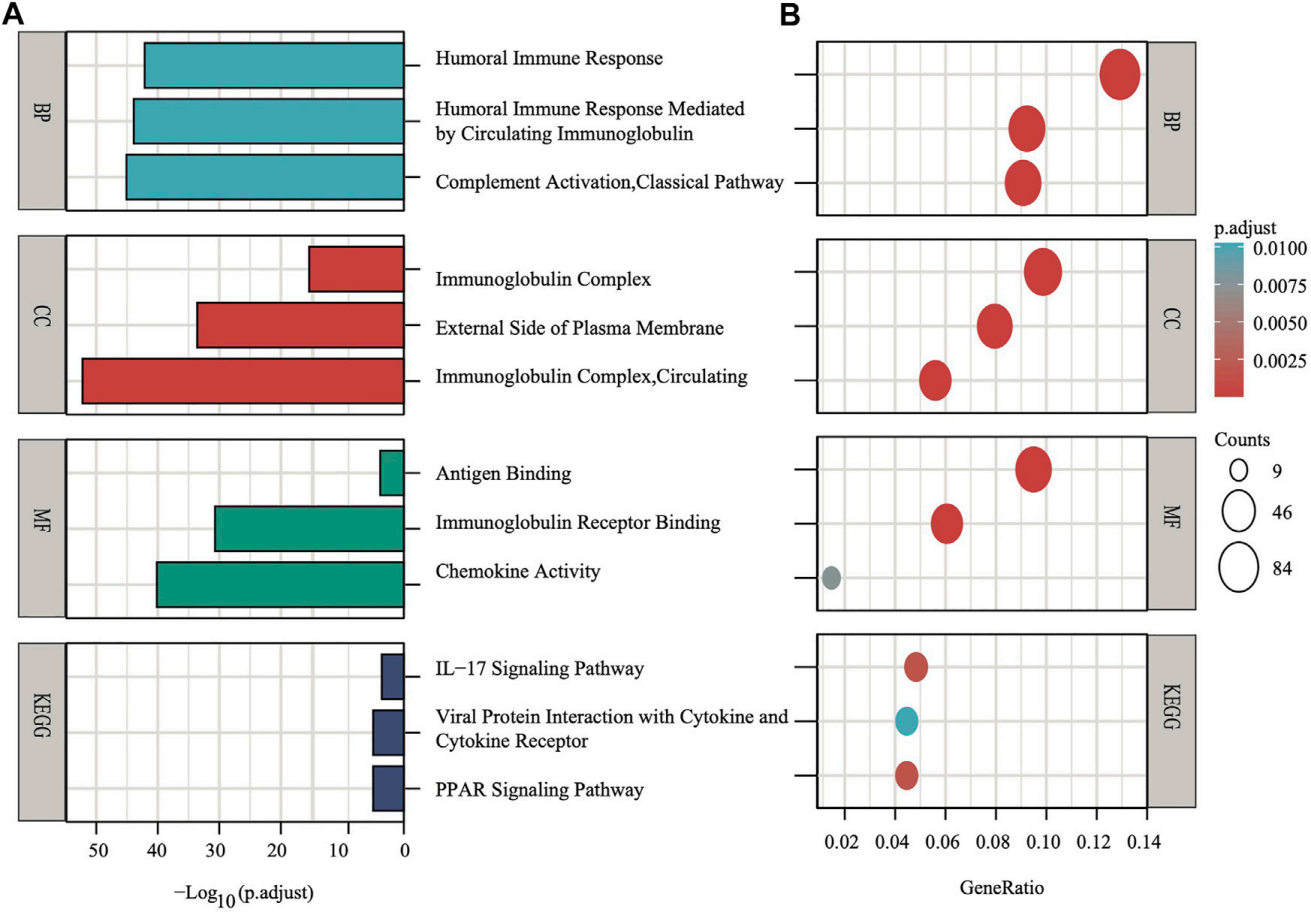
The *X*-axis depicts adjust *p* values, whereas the *Y*-axis depicts the enriched mechanism or pathway **(A)**. The *X*-axis depicts the gene ratio in the overall number of differential expression genes between two subgroups, whereas the *Y*-axis depicts the enriched mechanism or pathway **(B)**.

To explore the preliminary function of the nine-gene panel, KEGG pathway enrichment and GO function analyses were performed to compare the two stratified subgroups by running the ClusterProfiler R package (adjust *p* < 0.05, |logFC| > 1). The GO analysis showed that the genes were significantly enriched in immune-related functions (Figures 5A,B), such as humoral immune response, circulating immunoglobulin-mediated human immune response, complement activation, and classical pathways. These pathways functioned in antigen binding, immunoglobulin receptor binding, and chemokine activity. Furthermore, the KEGG analyses showed that the genes were enriched in the IL−17 signaling pathway, viral protein interaction with cytokine and cytokine receptor, and PPAR signaling pathway. IL−17 signaling transduction could regulate PD-1/PD-L1 and the infiltration of CD8^+^ T cells in patients with BRCA (Shuai et al., 2020), while the PPAR signaling pathway is activated in patients with TNBC (Lin et al., 2021). The PPAR pathway genes from KEGG analysis differentially expressed between two subgroups. Compared with the low risk subgroup, the expression of *MMP1, FABP5, FADS2, FABP7*, and *ME1* were upregulated, *UCP1, PLIN5, ADIPOQ, PLIN4, FABP4*, and *SLC27A2* were downregulated in high risk subgroup (Supplementary Figure S4). Study found that activating *MMP1* expression could increases multi-drug resistance in breast cancer (Shen et al., 2017). The *FADS2* activity associated with the aromatase drug letrozole in breast cancer cells (Park et al., 2021). The GO and KEGG analyses both showed that all pathways were immune-related.

#### Analysis of Immune Cell Enrichment

**FIGURE 6:**
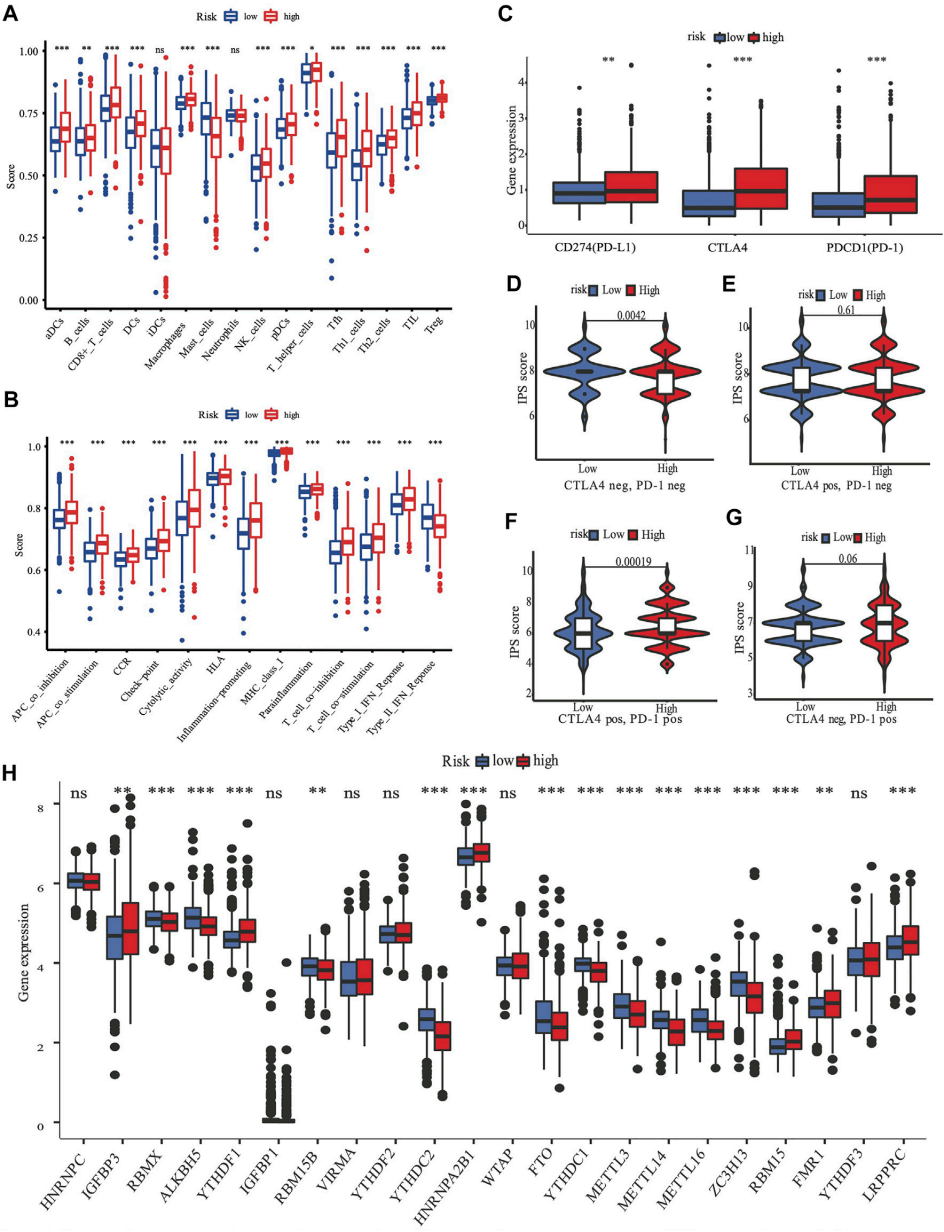
The role of nine-gene panel in immunotherapy based on TCGA cohort. Boxplots depict the scores of 16 immune cells **(A)** and 13 immune-related roles **(B)** in high and low risk subgroup by“ssGSEA”. Expression of immune checkpoints among high and low subgroups, such as CTLA4, PD-L1, PD-1 **(C)**; The immunophenoscore (IPS) distribution was also compared between high and low risk subgroups **(D–G)**. The gene expression levels of 22 m6A from TCGA cohort between two subgroups **(H)**. (ns, not significant; *p < 0.05; **p < 0.01; ***p < 0.001; pos means positive; neg means negative).

ssGSEA was used to quantify the scores of various immune cell subpopulations that corresponded to functions and pathways to further investigate the relationship between the nine-gene panel and immune status. Our findings revealed that the types of immune cells were significantly different between the two subgroups (Figures 6A,B). The high-risk subgroup had a significantly higher score than did the low-risk subgroup for most immune cells, including aDCs, CD8^+^ T, B, dendritic cells, natural killer cells, macrophages, plasmacytoid dendritic cells, T helper, Tfh, Th1 cells, Th2, TIL, and Treg cells, except for the score of mast cells, which was lower (Figures 6A,B). Furthermore, the high-risk subgroup had significantly higher scores for immune functions than did the low-risk subgroup. The immune functions were the check point, type I interferon response, T cell co-stimulation/inhibition, major histocompatibility class I, inflammation promotion, HLA, cytolytic activity, cytokine-cytokine receptor, parainflammation, and antigen presenting cell co-inhibition/stimulation (*p* < 0.05, Figures 6A,B). Analyses of both the TCGA and the METABRIC cohorts demonstrated that the results of the ssGSEA, GO, and KEGG analyses were consistent with the different immune functions and pathways of the two subgroups.

#### Analysis of m6A Methylation Regulators

A review summarized m6A methylation (Zaccara et al., 2019) regulators that we used for analysis, with 8 writers (*VIRMA, ZC3H13, WTAP, METTL14, METTL16, METTL3, RBM15B,* and *RBM15*), two erasers (*ALKBH5* and *FTO*), and 13 readers (*YTHDC1, YTHDC2, YTHDF1, YTHDF3, HNRNPC, FMR1, IGFBP3, RBMX, IGFBP1, YTHDF2, HNRNPA2B1, LRPPRC,* and *KIAA1429*). We found substantial variations in the expression of m6A regulators between high and low risk subgroups (Figure 6H). Compared with the low risk subgroup, the expression of *IGFBP3,YTHDF1, HNRNPA2B1, RBM15, FMR1,* and *LRPPRC* were upregulated, *RBMX, ALKBH5, RBM15B, YTHDC2, FTO, YTHDC1, METTL3, METTL14, METTL16,* and *ZC3H13* were downregulated in the high risk subgroup.

### The Risk Score Was Characterized by Distinct Immunotherapy Landscapes Circumstance

Targeting the immunological checkpoints CTLA4, PD-L1, and PD-1, has made significant progress in recent years in Her2-positive and TNBC patients. Our results showed that the high-risk subgroup was closely associated with Her2-positive and TNBC subtypes (Figure 4C; Table 2). As a result, we looked at the differences in immunological checkpoint expression between the two subgroups. The results showed that patients in the high-risk subgroup displayed a high abundance of PD-1, CTLA4, and PD-L1 (Figure 6C). Given the significant connection between the risk subgroups and immunological response, the response to ICIs treatment represented by CTLA4/PD-1 inhibitors was further examined in terms of immunotherapy across the two subgroups. Patients in the high-risk subgroup showed higher ICIs scores than those in the low-risk subgroup, when the CTLA4 and PD-1 statuses were both positive or negative (Figures 6D–F). This indicated that patients in the high-risk subgroup, whose CTLA4 and PD-1statuses were both positive or negative, demonstrated a substantial clinical benefit from combination therapy with anti-CTLA4 and anti-PD-1. Our results, taken together, clearly indicate that the nine-FRG panel is linked to immunotherapy response.

## Discussion

Cell death plays an essential role in the homeostasis of the body. An advantage of cell death is the prevention of the uncontrolled growth and proliferation of cancer cells, which have excessive energy demands to maintain their infinite self-renewal potential. Cancer is associated with alterations in energy metabolism, antioxidants, and intake of iron (Stockwell et al., 2017). Therefore, tumor cells are more vulnerable to iron-induced necrosis, which is also known as ferroptosis, due to their iron-dependent growth mechanism (Dixon et al., 2012).

A few study developed a similar model to predict BRCA prognosis and validated the FRG expression level using cell lines (Zhu et al., 2021a; Wu et al., 2021). However, it did not consider the molecular subtype and was not based on DSS. Further, only three genes in that model (*G6PD, FLT3*, and *SLC1A4*) overlapped with those in our DSS model. After the multivariate Cox regression analysis, the ferroptosis-related nine-gene panel that we discovered demonstrated an excellent prognostic prediction capability in the TCGA, METABRIC, and GSE3494 cohorts. The high-risk subgroup was significantly associated with a high tumor grade, an advanced TNM stage, and TNBC and Her2-positive subtypes, which were all related to poor survival and refractory treatment response. Conversely, the low-risk subgroup was positively associated with positive statuses of ER and PR and a negative HER2 status (Table2), which all corresponded to favorable survival outcomes in the traditional classification. Due to the high recurrence and metastatic rate, Her2-positive BRCA and TNBC have been regarded as refractory and aggressive subtypes (Foulkes et al., 2010; Cesca et al., 2020). Hence, researchers are focusing on exploring more therapeutic methods to fill this gap. Several studies suggested that targeting ferroptosis may be a useful therapeutic approach in the treatment of TNBC and Her2-positive BRCA (Chen et al., 2017; Nagpal et al., 2019; Zhu et al., 2020; Ding et al., 2021; Zhang et al., 2021).

The predictive capability of the nine-gene panel was more significant than those of traditional indicators (Figure 4A) in terms of tumor size, lymph node metastasis, and ER/PR and HER2 statuses, especially for TNBC and Her2-positive BRCA subtypes. This finding suggested that treatment could be escalated or de-escalated depending on patients’ risk stratification combined with canonical methods. However, the relationship between the nine genes and ferroptosis needs further exploration. The median follow-up of the TCGA was 2.5 years when the events was only 8% (Table 1), while METABRIC and GSE3494 cohorts were about 10 years, resulting in the AUC value for 3 years and 5 years DSS prediction is lower than the 2 years in TCGA. The nine-gene panel was a more reliable predictor of 2 years DSS in the TCGA (AUC = 0.806), METABRIC (AUC = 0.706), and GSE3494 cohorts (AUC = 0.624) (Figures 3D–F) because it had a high predictive accuracy for DSS, which was even better than those of the statuses of ER, PR, HER2, and lymph nodes as well as tumor size (Figures 4A,B).

**FIGURE 7:**
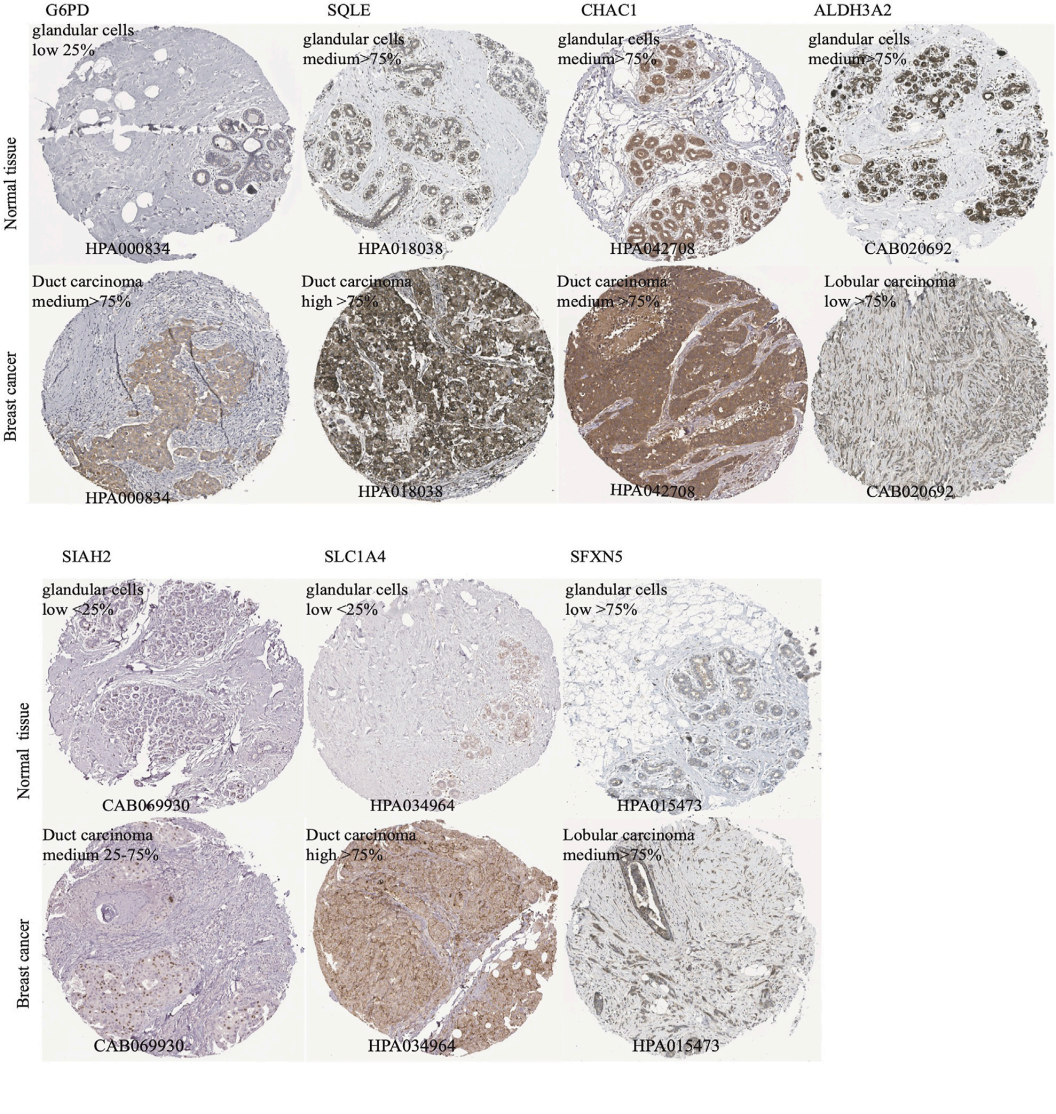
The immunohistochemistry (IHC) of ferroptotic prognostic protein images from the HPA database. The G6PD, SQLE, CHAC1, ALDH3A2, SIAH2, SLC1A4, and SFXN5 proteins level in breast cancer and adjcent normal tissues were shown by the IHC staining.

A total of nine FRG constituted our panel. These FRG were *ALDH3A2, SIAH2, G6PD, SLC1A4, FLT3, SQLE, EGLN2, SFXN5*, and *CHAC1*. *G6PD, SQLE*, and *CHAC1* were unfavorable genes for BRCA prognosis in this gene panel, and they were significantly overexpressed in the tumor compared with levels in the adjacent tissues (Figures 2B, 7). Contrastingly, the other genes were protective. A few studies have discovered that these nine genes are all associated with ferroptosis. The *SQLE* gene encodes squalene epoxidase to catalyze the oxidation of squalene that could change the lipid profile of tumor cells and protect them from ferroptosis (Garcia-Bermudez et al., 2019). In addition, it is one of the most significantly upregulated genes in many tumors, especially in BRCA (Xu et al., 2020). Moreover, Qin et al. (Shen et al., 2017) found that *SQLE* mRNA is stabilized by lnc030 in collaboration with poly (rC) binding protein 2(PCBP2), resulting in an increase in cholesterol synthesis. They suggested that targeting *SQLE* might be a potential mechanism of terbinafine in the treatment of BRCA (Qin et al., 2021). The *G6PD* gene encodes glucose-6-phosphate dehydrogenase, which produces NADPH to keep the glutathione (GSH) in balance to inhibit ferroptosis. In addition, *G6PD* eliminates ROS (Hao et al., 2018). Hence, the upregulation of *G6PD* facilitates cancer development and is thus associated with a poor prognosis in many forms of carcinomas (Ju et al., 2017; Chen et al., 2018; Zhu et al., 2021b). Likewise, the upregulated expression of *CHAC1* could prognosticate unfavorable outcomes in BRCA (Goebel et al., 2012; Li et al., 2021). Additionally, *CHAC1* has been discovered to decease intracellular GSH levels, enhancing tumor cell ferroptosis (Chen et al., 2017). Moreover, *FLT3, ALDH3A2,* and *SIAH2* gene depletion or deficiency can result in ferroptosis (Kang et al., 2014; Hassannia et al., 2019; Chillappagari et al., 2020). A loss in *ALDH3A2* triggers ferroptosis and cooperates with *GPX4* inhibition (Yusuf et al., 2020). *SIAH2*-deficient cells exhibit increased vulnerability to ferroptosis, and re-expression of *GPX4* can rescue these cells from ferroptosis (Chillappagari et al., 2020). Equally, *EGLN2* knockdown inhibited ferroptosis in mice, researchers validated the mRNA level of prostaglandin-endoperoxide synthase two b y qPCR, it is as a marker for assessing ferroptosis *in vivo*; and *EGLN2* gene could mediate *HIF1A* downregulation to promote ferroptosis (Yang et al., 2019). Only few researches have focused on *SLC1A4* and *SFXN5*. However, they did not find an association between these genes and ferroptosis. Nonetheless, *SLC1A4* may promote ferroptosis and may function as a marker of ferroptosis, according to the FerrDb website data. Moreover, *SFXN5* may be involved in cellular iron ion homeostasis (Tifoun et al., 2021). These two genes need further study to confirm their relationship with ferroptosis. Further, these genes are all linked to the promotion or prevention of ferroptosis in various cancers *via* multiple mechanisms (Supplementary Figure S3); however, it is unclear whether these genes influence the prognosis of BRCA patients through ferroptosis.

Recently, researchers have been exploring the role of ferroptosis in tumor therapy. However, the possible regulation of tumor immunity and ferroptosis remains a mystery. The idea that immunity stimulates or inhibits cancer cells is widely known, and targeting the immune checkpoint has become a promising and potential therapeutic approach in recent years. Studies that involved the TCGA and METABRIC cohorts revealed that the majority of the types of immune cells in the high-risk subgroup had higher immune scores than did those in the low-risk subgroup, except for mast cells (Figure 6A). Furthermore, the high-risk subgroup also had excessively higher scores for most immune functions (Figure 6B). There may be a crosstalk between the ferroptosis of cancer cells and those of infiltrating immune cells. One theory is that ferroptotic cancer cells emit distinct signals, which cause phagocytosis and induce antigen presentation by dendritic cells (Friedmann Angeli et al., 2019). Meanwhile, the suppression of ferroptotic activity impairs the capacity of CD8^+^ T and natural killer cells to destroy cells *in vivo* (Wang et al., 2019).

The results of the GO analysis suggested that many immune-related pathways and biological processes, such as humoral immune response, circulating immunoglobulin-mediated human immune response, complement activation, and classical pathways were enriched. The enriched KEGG pathways were the IL−17 signaling pathway and the PPAR signaling pathway (Figures 5A,B). The IL−17 signaling transduction regulated the infiltration of CD8^+^ T cells and PD-1/PD-L1 in BRCA patients (Shuai et al., 2020), while the PPAR signaling pathway was activated in TNBC patients (Lin et al., 2021). Our results demonstrated that the TNBC subtypes (91% in TCGA, 98.7% in METABRIC, Figure 4C) were mostly found in the high-risk subgroup (Table2). The clinical trial of combination anti-PD-1/PD-L1 showed that the progression-free survival was significantly improved in TNBC patients with metastasis (Cortes et al., 2020). The mechanism of anti-PD-L1 antibodies was to trigger ferroptosis, which subsequently enhanced the efficacy of immunotherapy (Wang et al., 2019). The resistance to anti-PD-1/PD-L1 in TNBC cells inhibited ferroptosis and changed the proportion of macrophage cells (Jiang et al., 2021). Therefore, ferroptosis may be induced through changes in the immune system. According to our panel, BRCA patients are stratified into the high- and low-risk subgroups. The patients in the high risk subgroup, whose CTLA4 and PD-1statuses were both positive or negative, demonstrated a substantial clinical benefit from combination therapy with anti-CTLA4 and anti-PD-1 (Figures 6D,F). Therefore, the high-risk subgroup should be administered intensive treatment or immunotherapies, whereas the low-risk subgroup should be administered de-escalated treatment. The relationship between immunity and ferroptosis has not been thoroughly clarified. However, there might be a strong relationship between the immune microenvironment of the tumor and ferroptosis in BRCA patients. Thus, further research is needed to validate the above findings.

Our study has several limitations. First, since ferroptosis research is a new and rapidly expanding field, more FRG are likely to be discovered in the future. Second, because of the observed heterogeneity between different populations, the findings of this retrospective and cross-cohort research need validation by further prospective reviews involving multicenter cohorts. Third, since the data were downloaded from public databases, several important clinical details were not accessible. These inaccessible data included chemotherapy regimens, drug information, and tumor burden, and the lack of these data limited a more in-depth comparison among the TCGA, METABRIC, and GSE3494 data. Finally, since the results are based on RNA sequence, verification of protein expression in terms of immunohistochemistry is needed to conveniently apply our findings in clinical practice.

## Conclusion

We constructed a DSS prognostic prediction panel involving nine FRG genes in BRCA. This panel was based on FRG and DSS events. In the TCGA, METABRIC, and GSE3494 cohorts, our panel was independently correlated with DSS prognosis. We also found that the tumor immune microenvironment and ferroptosis may have a strong inherent connection with BRCA. This panel could be used to evaluate prognosis and to select patients for escalation/de-escalation treatment. Our research provides a preliminary theory for clinically individualized therapy by targeting ferroptosis genes.

## Data Availability

Publicly available datasets were analyzed in this study. This data can be found here: https://portal.gdc.cancer.gov/repository http://www.cbioportal.org/study/summary?id=brca_metabric https://www.ncbi.nlm.nih.gov/gds/?term=GSE3494.
