# Supplementary figures and images for: Development and Validation of a Robust Ferroptosis-Related Gene Panel for Breast Cancer Disease-Specific Survival
Source: Front Cell Dev Biol. 2021 Nov 25;9:709180. doi: 10.3389/fcell.2021.709180 (PMC8655913; doi:10.3389/fcell.2021.709180)

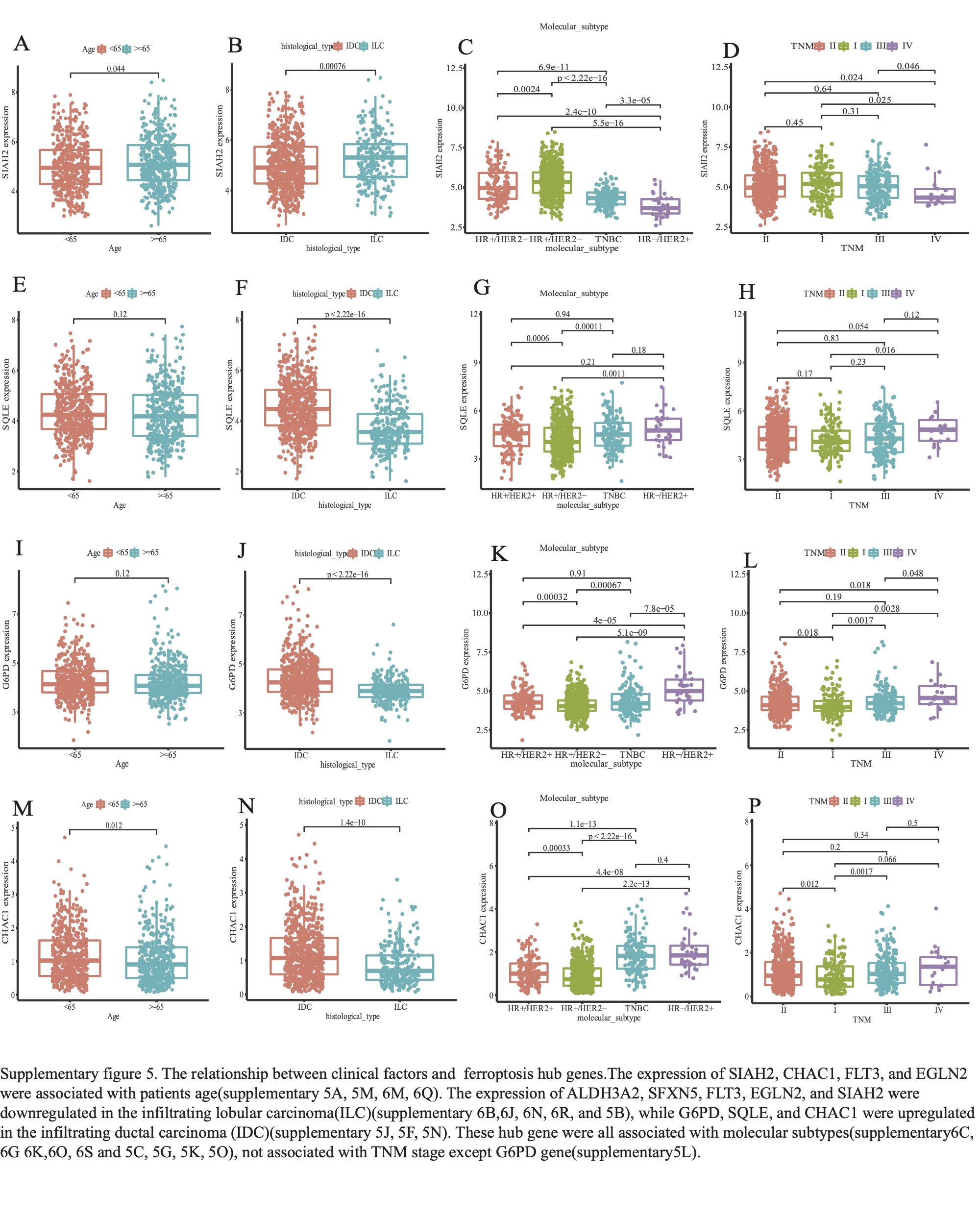

Supplement: Supplementary file 2 [file Image3.JPEG]

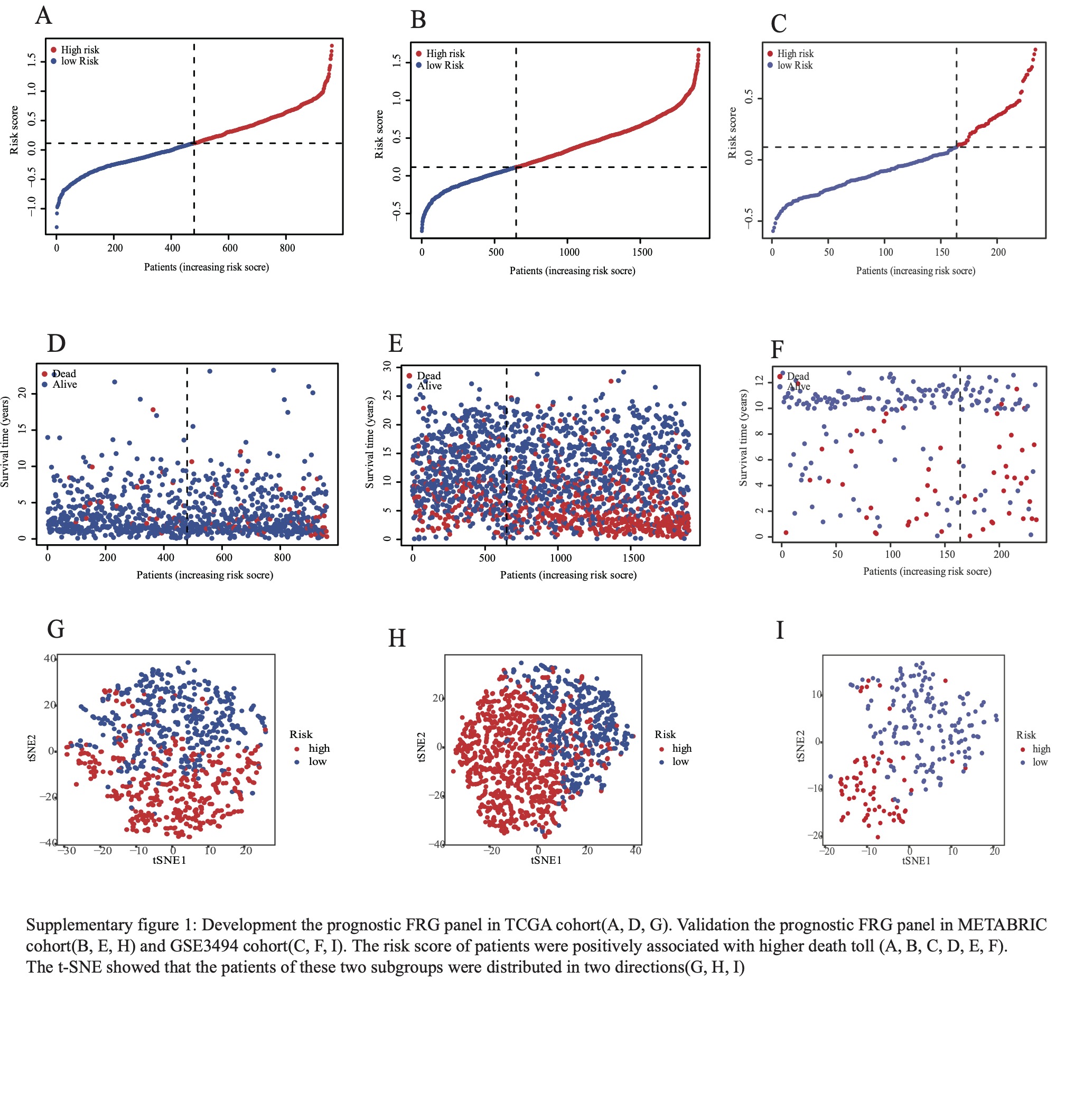

Supplement: Supplementary file 3 [file Image1.JPEG]

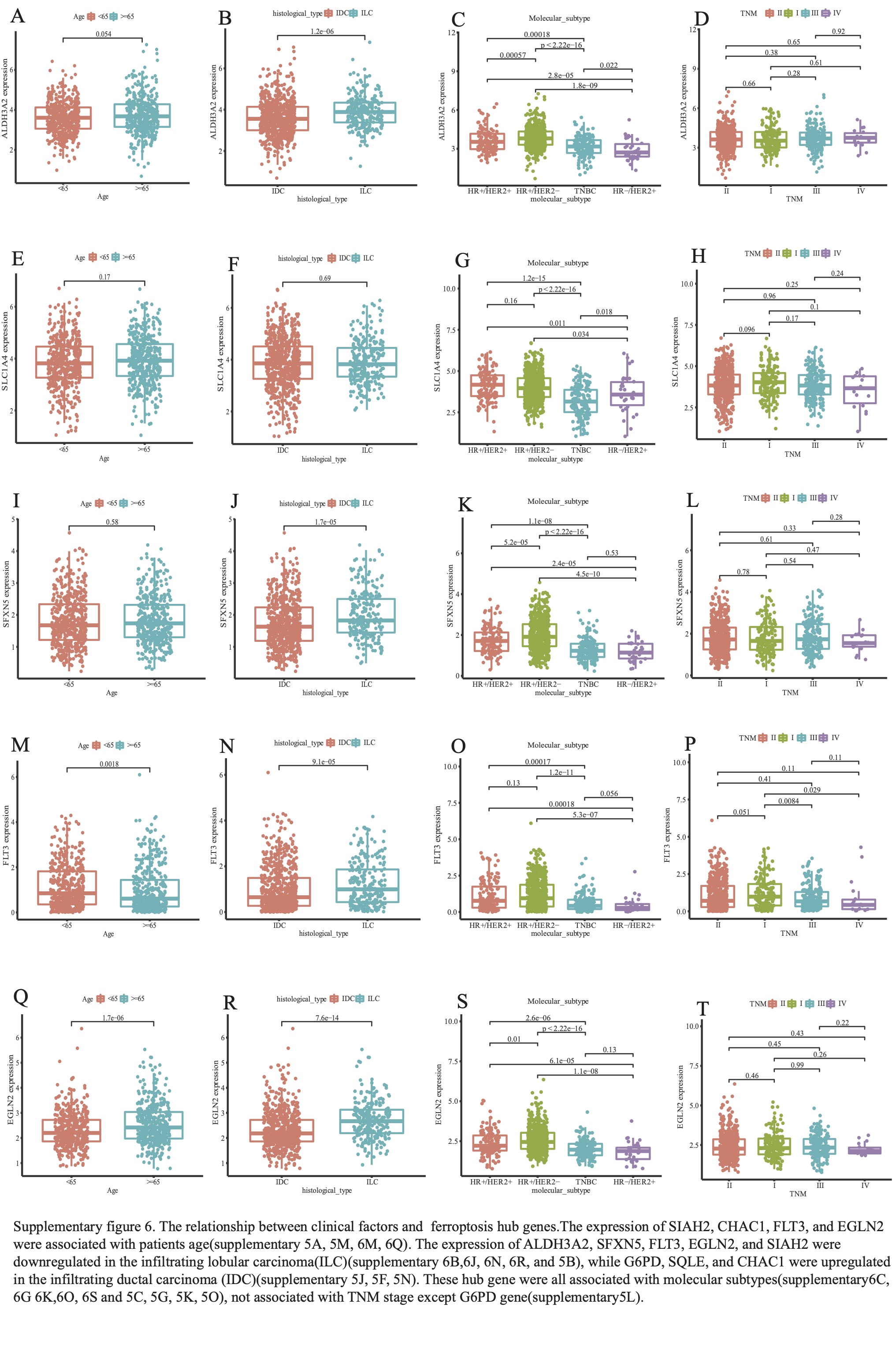

Supplement: Supplementary file 4 [file Image4.JPEG]

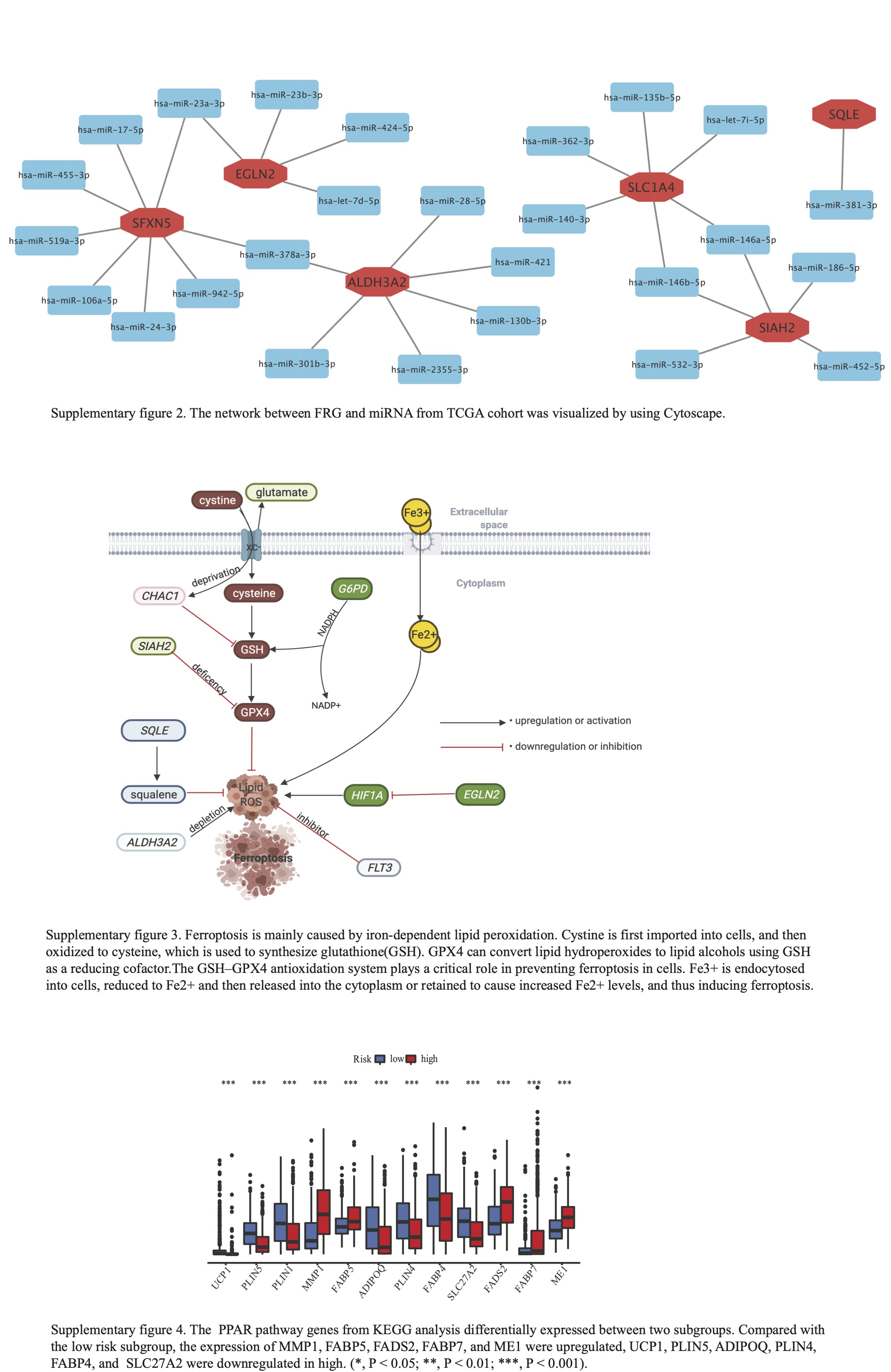

Supplement: Supplementary file 5 [file Image2.JPEG]
